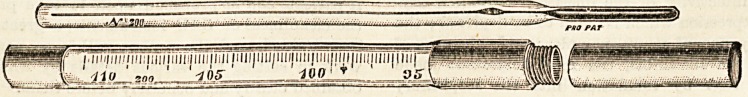# New Appliances and Things Medical

**Published:** 1904-02-13

**Authors:** 


### NEW APPLIANCES AND THINGS MEDICAL.

[We shall be glad to receive at our Office, 28 & 29 Southampton Street, Strand, London, W.C., from the manufacturers, specimens of all new preparations and appliances which may be brought out from time to time.]

### SAEFINUSE THERMOMETER.

(MESSRS. S. MAW, SON AND SONS, 7–12 ALDERSGATE STREET, LONDON, E.C.)

THERE is no doubt that the ordinary clinical thermometer a possible source of danger in the matter of conveying infection unless scrupulous care be taken. It is with the object of minimising such risk that this instrument has been placed on the market. The apparatus, as shown in the accompanying illustration, consists of an outer glass cylinder on which is marked the temperature scale, and a perfectly smooth clinical thermometer which is placed within the glass casing when the temperature is read. The advantages of such an arrangement are considerable. The smooth thermometer can be easily cleaned and disinfected, while the scale markings, being on the outer casing, are not liable to become obliterated by frequent washing. The glass cylinder may be easily rendered sterile by placing in it a little antiseptic solution. There is another point which may be claimed in favour of this instrument, viz., that the patients are unable to read their own temperatures, a matter which may be of no inconsiderable importance in some cases. On testing the instrument submitted to us we find that perfectly accurate readings are obtained.

### "PHARMA" MILK FOOD.

(JAMES TAYLOR, 132 TRONGATE, GLASGOW.)

EXAMINATION of this food shows that it closely approaches the standard of composition to which artificial substitutes for human milk should conform. The percentage of fat is fairly high and the carbohydrate elements are not too rich in proportion, the reverse of which is the fault so commonly found to exist in preparations of this kind. This food is quickly prepared, for it only requires the addition of hot water to be ready for use. The palatability and easy assimilation of "Pharma" Food will commend its use to invalids of maturer years as well as to infants.

## Figures and Tables

**Figure f1:**